# Harm perceptions of e‐cigarettes and other nicotine products in a UK sample

**DOI:** 10.1111/add.14502

**Published:** 2019-01-03

**Authors:** Samara Wilson, Timea Partos, Ann McNeill, Leonie S. Brose

**Affiliations:** ^1^ Addictions Department, Institute of Psychiatry, Psychology and Neuroscience King's College London London UK; ^2^ UK Centre for Tobacco and Alcohol Studies UK

**Keywords:** Electronic cigarettes, electronic nicotine delivery systems, harm perception, harm reduction, nicotine replacement products, smoking, vaping

## Abstract

**Background and aims:**

E‐cigarettes (EC) and nicotine replacement therapy (NRT) are less harmful than smoking, but misperceptions of relative harm are common. Aims were to (1) assess nicotine knowledge and perceptions of: harm of EC and NRT relative to smoking, addictiveness of EC relative to smoking, and change in harm to user if smoking replaced with EC; (2) define associations of these perceptions with respondent characteristics including nicotine knowledge; and (3) explore perceived main harms of EC and whether these differ by vaping status.

**Design:**

Analyses were: (1) frequencies; (2) logistic regressions of perceptions of relative harm, addictiveness and change in harm onto demographics, smoking and vaping status and nicotine knowledge (attributing cancer or health risks of smoking to nicotine); and (3) frequencies and χ^2^ statistics.

**Setting and participants:**

Participants were smokers and recent ex‐smokers from one wave (September 2017) of a longitudinal online survey in the United Kingdom (*n* = 1720).

**Measurements:**

Demographics included gender, age, smoking status, vaping status and income. Survey questions collected data on nicotine knowledge and harm perceptions of different products; the relative harm perceptions of NRT, EC and tobacco cigarettes; and perceived main harms of EC.

**Findings:**

Relative to smoking, 57.3% perceived EC and 63.4% NRT to be less harmful; 25.4% perceived EC to be less addictive; and 32.2% thought replacing smoking with EC reduced health harms a great deal. Participants were less likely to endorse these beliefs if they had never vaped, and participants who had inaccurate nicotine knowledge were less likely to endorse all these beliefs apart from the addictiveness of EC. The main concerns about EC were a lack of research (48.3%), regulation or quality control (37.8%) and harmfulness of chemicals (41.6%).

**Conclusions:**

Large proportions of UK smokers and ex‐smokers overestimate the relative harmfulness of e‐cigarettes and nicotine replacement therapy compared with smoking; misattributing smoking harms to nicotine is associated with increased misperceptions.

## Introduction

Smoking tobacco cigarettes is one of the most dangerous behaviours, as it kills more than half of those with sustained use. There are many health harms from smoking, such as increased risk of heart attack, stroke and many cancers. Nicotine is the main addictive substance in smoking, but it is the other constituents that cause the harms 1. Nicotine is also used in nicotine replacement therapy (NRT), which does not have the same level of health risks associated with smoking. For example, the Royal College of Physicians report 2 found no increased risk of heart attack, stroke or death from using NRT when attempting to quit smoking. The Lung Health study 3, a 5‐year randomized controlled trial (RCT) with a 7.5‐year follow‐up, found no link between NRT and cancers, whereas there was a relationship between smoking and cancer. Electronic cigarettes (EC) can also contain nicotine and are less harmful than smoking 2, 4, 5, 6; however, there are many misperceptions in the community concerning the level of harm presented by nicotine, and the relative harm of smoking tobacco cigarettes versus NRT and EC 4, 5, 7, 8. It is possible that misperceptions of nicotine's risks are underpinning harm perceptions of NRT and EC, but evidence on this potential association is lacking.

It is currently unclear how harm misperceptions of different products affect their use, as findings have been mixed. Brose *et al*. 9 looked at harm perceptions of EC among past‐year smokers, and found that perceiving EC to be less harmful predicted subsequent initiation of EC use. However, Black *et al*. 10 did not find a link between accurate nicotine knowledge and NRT usage.

Both internationally and among adults and youth in Great Britain, harm perceptions of nicotine and EC were not improving over time 4, 11, 12, 13, 14. The 2014 Tobacco Products Directive (revised EU TPD) placed regulations on EC, such as restricting nicotine concentration and the size of tanks and refill bottles 4. These regulations, which became applicable in the UK between 20 May 2016 and 20 May 2017, could potentially influence smokers into believing EC are more harmful than they are, or could reassure smokers about the safety of the EC products on the market after EU TPD implementation. This makes current harm perceptions highly relevant to investigate.

This study used a web‐based national sample of past‐year smokers from the general population in the UK and aimed to (1) assess nicotine knowledge and perceptions of harm of EC and NRT relative to smoking, addictiveness of EC relative to smoking and change in harm to user if smoking replaced with EC; (2) associations of these perceptions with respondent characteristics including nicotine knowledge; and (3) explore perceived main harms of EC and whether these differ by vaping status.

## Methods

### Participants and design

Data from a UK longitudinal online survey were used. Members of an online panel managed by Ipsos MORI were invited by e‐mail to participate in a study about smoking by Ipsos Interactive Services in return for points, which could be redeemed as shopping vouchers or used to enter prize draws. At baseline, of the 23 785 invited participants, 6165 were eligible to participate, being past‐year smokers, i.e. reported that they smoked cigarettes (including hand‐rolled) every day or some days, smoked tobacco of some kind (e.g. pipe or cigar) or had stopped smoking completely in the past year 15. To ensure representativeness of the British population, quotas were imposed based on age, gender and region at recruitment. In November and December 2012, 5000 participants completed the baseline/wave 1 survey, 2182 of the original participants completed the survey in wave 2 (December 2013) and 1519 in wave 3 (December 2014). In wave 4 (May/June 2016), the sample was replenished using the same approach as for wave 1, with quotas again imposed based on age, gender and region, resulting in 3334 survey completions (*n* = 931 continuing from wave 1 and *n* = 2403 newly recruited). At wave 5 (September 2017), 1720 participants completed the survey. This current paper analyses data from wave 5.

### Measures

#### Demographics

Demographic variables included gender (male or female) and age. Smoking status (daily, non‐daily, other tobacco, recent quit, quit more than 1 year ago) was recoded into daily smoker, non‐daily smoker and ex‐smoker. Vaping status (daily, non‐daily, only a few times, recent quit, quit more than 1 year ago, never vaped) was recoded into daily vaper, non‐daily vaper, trier, ex‐vaper and never vaped. Income was grouped as low (under £15 000), moderate (£15 001–30 000), high (more than £30 000) or not disclosed. The wording of the measures is provided in Supplementary table 1.

#### Nicotine knowledge and harm perceptions of different products

Four items from the survey assessed relative harm perceptions of NRT, EC, nicotine and tobacco cigarettes, and two items assessed nicotine knowledge. Binary recoding categories were selected in which one category was deigned accurate based on the Royal College of Physician's report on tobacco harm reduction (2016).

Items for nicotine knowledge were:

- ‘According to what you know or believe, what portion of the health risks of smoking comes from nicotine in tobacco cigarettes?’ Response options were: ‘none or very small; some but well under half the risk; around half the risk; much more than half the risk; nearly all the risk; don't know’. This was recoded as a binary variable with two levels: none or very small risk (the correct answer) versus all other response options.
- ‘The nicotine in tobacco cigarettes is the chemical that causes most of the cancer.’ Response options were ‘true’ or ‘false’. False was the correct answer.

Items for the relative harm perceptions of NRT, EC and tobacco cigarettes were:

- ‘Do you think electronic cigarettes/vaping devices are more harmful than smoking tobacco cigarettes, less harmful, or are they equally harmful to health?’ Response options were: ‘more harmful than tobacco cigarettes; equally harmful; less harmful than tobacco cigarettes; don't know’. This was dichotomized into ‘EC are less harmful than tobacco cigarettes’ (correct answer) versus all other responses.
- ‘Do you think nicotine replacement therapies such as gums or patches are more harmful than smoking tobacco cigarettes, less harmful, or are they equally harmful?’ Response options were the same as the previous item. This was dichotomized as NRT is less harmful than tobacco cigarettes (correct answer) versus all other categories.
- ‘Do you think electronic cigarettes/vaping devices (with nicotine) are more addictive than tobacco cigarettes, less addictive, or are they equally addictive?’ Response options were: ‘more addictive than tobacco cigarettes; equally addictive; less addictive than tobacco cigarettes; don't know’. This variable was dichotomized as less addictive versus all other responses.
- ‘Now imagine a smoker who smokes 10 tobacco cigarettes a day stops smoking tobacco cigarettes altogether and continues to use electronic cigarettes or vaping devices daily. How would that change any harm to their health from smoking and vaping?’ Response options were: ‘would reduce health harms a lot; would reduce health harms a little; would not change harm at all; would increase health harms a little; would increase health harms a lot; don't know’. This was dichotomized as would reduce health harms a lot versus all other response options.

#### Perceived main harms of EC

Participants were asked: ‘What in your view are the main harms, if any, of EC/vaping devices?’, followed by 11 options with yes/no responses: ‘there are no harms; they may be addictive; they may explode or catch fire; they may cause breathing/respiratory problems; they may cause cancer; there has not been enough research done to understand all the possible harms; the chemicals in the liquid might be harmful; the nicotine; there is not enough quality control or regulation—you don't know exactly what chemicals they may contain; they reinforce the smoking habit; they may cause heart disease’. These items represented a summary of concerns provided by participants in wave 4 to an open response item which asked about the main harms of EC/vaping devices.

### Data analysis

Data were analysed using Stata version 15. The analyses involved a cross‐sectional design using wave 5 data. Alpha was set at 0.05. An attrition analysis examined differences between respondents followed‐up at wave 5 versus those from wave 4 lost to attrition using Pearson's χ^2^ analyses.

### Aim 1: Nicotine knowledge and relative harm perceptions of EC, NRT and smoking

Frequencies to the responses to the two nicotine knowledge items and the four harm perception items were calculated.

### Aim 2: Associations of relative harm perceptions with respondent characteristics including nicotine knowledge

Bivariate and multivariable logistic regressions were used to assess associations between harm perception and demographics, smoking and EC use status and nicotine knowledge.

Results from multivariable analyses are reported in the table, and where bivariate results markedly differed this is noted in text.

### Aim 3: Perceived main harms of EC

Frequencies of the 11 items on the perceived main harms of EC were calculated, and a χ^2^ analysis was conducted to compare whether these frequencies differed based on vaping status. Adjusted standardized residuals (greater than 2 and 3 standard deviations marked) were used to explore where these differences were among the vaping status categories.

## Results

### Demographics

Our sample had a mean age of 49.4 years; other demographic, smoking and vaping characteristics are described in Table 1. Respondents followed‐up at wave 5 versus those lost to attrition were significantly more likely to be older age groups (χ^2^_(5)_ = 184.56, *P* < 0.001), more likely to have never vaped and less likely to have vaped only a few times (χ^2^_(5)_ = 34.66, *P* < 0.001), less likely to be non‐daily smokers (χ^2^_(5)_ = 27.50, *P* < 0.001) and less likely to have a high income (χ^2^_(3)_ = 17.10, *P* = 0.001). They did not differ on gender.

**Table 1:** Sample description, n = 1720.

Characteristics (wave 5)		% (n)
Gender	Female	44.1 (758)
	Male	55.9 (962)
Age (years)	18–24	4.9 (85)
	25–34	14.4 (247)
	35–44	18.2 (313)
	45–54	24.5 (422)
	55–64	18.3 (314)
	65+	19.7 (339)
Smoking status	Daily smoker	51.4 (884)
	Non‐daily smoker	15.9 (274)
	Ex‐smoker	32.7 (562)
Vaping status	Never vaped	39.0 (670)
	Daily vaper	17.6 (302)
	Non‐daily vaper	12.4 (214)
	Trier	17.1 (294)
	Ex‐vaper	14.0 (240)
Income	Low (≤ £15 000)	20.9 (359)
	Moderate (£15 001–30 000)	29.9 (514)
	High (> £30 000)	40.1 (690)
	Not disclosed	9.1 (157)

### Nicotine knowledge

For the portion of health risk which comes from nicotine in cigarettes, 11.2% responded none or very small, 24.5% responded some but well under half, 21.8% responded approximately half the risk, 16.3% responded much more than half the risk, 11.5% responded nearly all the risk and 14.8% responded that they did not know. In adjusted analyses, being aged 55–64 or 35–44 years (versus 65+) and being a daily vaper or vaping trier (versus never vaper) were associated with higher odds of responding that a very small proportion of the health risk in cigarettes comes from nicotine (Table 2).

**Table 2:** Portion of health risk which comes from nicotine in cigarettes, and the odds of responding that nicotine in cigarettes is not what causes most of the cancer, predicted from demographics using logistic regression; % shows proportion endorsing each of the statements.

Demographics		(i) None/very small amount of health harms of tobacco cigarettes comes from nicotine					(ii) Nicotine in cigarettes is not what causes most of the cancer				
			Bivariate		Multivariable			Bivariate		Multivariable	
		%	OR (95% CI)	P	OR (95% CI)	P	%	OR (95% CI)	P	OR (95% CI)	P
Gender	Female	9.8	ref		ref		59.1	ref		ref	
	Male	12.3	1.29 (0.95–1.76)	0.10	1.25 (0.91–1.72)	0.18	62.1	1.13 (0.93–1.38)	0.21	1.10 (0.90–1.35)	0.35
Age (years)				**0.011**		**0.042**			**0.001**		**0.004**
	65+	7.4	ref		ref		55.2	ref		ref	
	55–64	14.3	**2.10 (1.26–3.52)**	**0.005**	**1.93 (1.14–3.27)**	**0.014**	63.1	1.39 (1.01–1.90)	0.041	1.31 (0.95–1.80)	0.10
	45–54	12.8	**1.84 (1.12–3.03)**	**0.016**	1.57 (0.94–2.62)	0.082	67.3	**1.67 (1.25–2.25)**	**0.001**	**1.53 (1.13–2.06)**	**0.006**
	35–44	13.4	**1.95 (1.16–3.28)**	**0.012**	**1.73 (1.01–2.96)**	**0.046**	63.3	**1.40 (1.02–1.92)**	**0.036**	1.27 (0.92–1.76)	0.14
	25–34	6.9	0.93 (0.49–1.76)	0.82	0.87 (0.45–1.67)	0.67	55.1	1.00 (0.72–1.38)	0.98	0.94 (0.67–1.32)	0.72
	18–24	10.6	1.49 (0.67–3.32)	0.33	1.27 (0.56–2.90)	0.57	49.4	0.79 (0.49–1.28)	0.34	0.71 (0.44–1.16)	0.17
Smoking status				**0.007**		0.16			0.086		0.24
	Daily smoker	9.4	ref		ref		59.7	ref		ref	
	Non‐daily	9.9	1.05 (0.67–1.67)	0.82	1.07 (0.67–1.72)	0.77	56.9	0.89 (0.68–1.17)	0.41	0.92 (0.69–1.23)	0.58
	Ex‐smoker	14.6	**1.65 (1.19–2.28)**	**0.003**	1.41 (0.98–2.03)	0.06	64.2	1.21 (0.97–1.51)	0.086	1.18 (0.93–1.49)	0.18
Vaping status				**< 0.001**		**< 0.001**			**< 0.001**		**< 0.001**
	Never vaped	7.2	ref		ref		54.9	ref		ref	
	Daily vaper	21.9	**3.62 (2.43–5.41)**	**< 0.001**	**3.23 (2.13–4.90)**	**< 0.001**	72.2	**2.13 (1.59–2.86)**	**< 0.001**	**1.97 (1.46–2.67)**	**< 0.001**
	Non‐daily vaper	6.1	0.84 (0.44–1.58)	0.59	0.89 (0.47–1.70)	0.72	53.3	0.94 (0.69–1.27)	0.67	0.97 (0.70–1.33)	0.85
	Trier	13.6	**2.04 (1.31–3.18)**	**0.002**	**2.20 (1.39–3.48)**	**0.001**	67.7	**1.72 (1.29–2.29)**	**< 0.001**	**1.78 (1.32–2.39)**	**< 0.001**
	Ex‐vaper	10.4	1.51 (0.91–2.50)	0.11	1.41 (0.84–2.37)	0.19	60.8	1.27 (0.94–1.72)	0.11	1.25 (0.92–1.70)	0.16
Income				0.94		0.95			0.39		0.58
	Low	10.3	ref		ref		57.9	ref		ref	
	Moderate	11.5	1.13 (0.73–1.74)	0.59	1.11 (0.71–1.73)	0.66	61.3	1.15 (0.87–1.51)	0.32	1.16 (0.88–1.54)	0.29
	High	11.5	1.13 (0.74–1.70)	0.58	1.00 (0.64–1.54)	0.99	62.6	1.22 (0.94–1.58)	0.14	1.16 (0.89–1.53)	0.28
	Not disclosed	10.8	1.06 (0.58–1.94)	0.86	1.04 (0.56–1.95)	0.90	57.3	0.98 (0.67–1.43)	0.90	0.99 (0.67–1.46)	0.95

OR = odds ratio; CI = confidence interval; *n* = 1720. (i) *R* ^2^ = 0.039 (Cox & Snell), 0077 (Nagelkerke). Model χ^2^_(15)_ = 67.80, *P* < 0.001. **(**ii) *R* ^2^ = 0.035 (Cox & Snell), 0.048 (Nagelkerke). Model χ^2^_(15)_ = 61.84, *P* < 0.001. Bold type = *P <* 0.05; ref = reference category.

When asked whether the nicotine in tobacco cigarettes is the chemical that causes most of the cancer, 39.2% responded true (the incorrect response) and 60.8% responded false (the accurate response). In adjusted analyses, being aged 45–54 years (versus 65+), a daily vaper or vaping trier (versus never vaper) were significantly associated with higher odds of endorsing that nicotine in cigarettes is not what causes most of the cancer (Table 2).

### Relative harm perceptions and addictiveness perceptions of EC, NRT and smoking, and associations with respondent characteristics

For the relative harmfulness of EC and tobacco cigarettes, 3.3% responded that EC were more harmful than tobacco cigarettes, 21.8% responded equally harmful, 57.3% responded less harmful and 17.6% responded they did not know. In the multivariable analysis, being in any of the age groups between 25–54 was associated with lesser odds, and being an ex‐smoker or having ever vaped were associated with greater odds for perceiving EC as less harmful than tobacco cigarettes versus all the other responses (Table 3). Bivariate analyses did not find a difference for ages 35–54.

**Table 3:** Multivariable logistic regressions predicted from demographics and nicotine knowledge for: (i) relative harm perception of EC versus tobacco cigarettes; (ii) relative harm perception of NRT versus tobacco cigarettes; (iii) responding EC are less addictive than tobacco cigarettes; and (iv) perceived change in harm if a smoker stopped smoking and used EC instead; % shows proportion endorsing each of the statements.

		(i) EC are less harmful than tobacco cigarettes			(ii) NRT are less harmful than tobacco cigarettes			(iii) EC are less addictive than tobacco cigarettes			(iv) Replacing smoking with EC would reduce health harms a great deal		
	Variable	%	OR (95% CI)	P	%	OR (95% CI)	P	%	OR (95% CI)	P	%	OR (95% CI)	P
**Demographics**													
Gender	Female	55.7	ref		64.6	ref		22.7	ref		32.9	ref	
	Male	58.5	1.03 (0.84–1.27)	0.78	62.5	0.83 (0.67–1.02)	0.079	27.6	**1.29 (1.02–1.63)**	**0.034**	31.6	0.86 (0.69–1.08)	0.19
Age (years)				**0.002**			**0.007**			0.40			**< 0.001**
	65+	59.9	ref		63.4	ref		25.1			35.1	ref	
	55–64	59.9	0.77 (0.55–1.08)	0.13	66.9	1.01 (0.73–1.42)	0.95	23.6	0.75 (0.52–1.10)	0.14	36.6	0.83 (0.59–1.16)	0.27
	45–54	58.3	**0.65 (0.47–0.89)**	**0.008**	67.1	0.99 (0.72–1.35)	0.94	24.6	0.78 (0.55–1.10)	0.16	33.7	**0.67 (0.49–0.93)**	**0.016**
	35–44	55.6	**0.55 (0.39–0.77)**	**0.001**	62.0	0.75 (0.54–1.05)	0.10	28.1	0.88 (0.60–1.27)	0.48	27.8	**0.50 (0.35–0.72)**	**< 0.001**
	25–34	49.8	**0.49 (0.35–0.71)**	**< 0.001**	53.0	**0.58 (0.40–0.82)**	**0.002**	23.5	0.71 (0.47–1.06)	0.10	23.9	**0.47 (0.31–0.69)**	**< 0.001**
	18–24	60.0	0.70 (0.41–1.17)	0.17	68.2	1.12 (0.66–1.91)	0.67	32.9	1.07 (0.62–1.85)	0.81	36.5	0.86 (0.50–1.45)	0.56
Smoking status				**0.003**			**0.032**			0.35			0.056
	Daily smoker	53.4	ref		62.6	ref		25.9	ref		31.3	ref	
	Non‐daily	55.8	1.15 (0.86–1.55)	0.35	58.0	0.85 (0.63–1.13)	0.27	22.3	0.78 (0.55–1.10)	0.16	24.8	0.73 (0.53–1.02)	0.066
	Ex‐smoker	64.1	**1.52 (1.19–1.94)**	**0.001**	67.4	1.27 (0.99–1.62)	0.056	26.2	0.98 (0.75–1.29)	0.91	37.0	1.13 (0.87–1.45)	0.36
Vaping status				**< 0.001**			**0.041**			**<0.001**			**< 0.001**
	Never vaped	43.0	ref		59.6	ref		13.1	ref		21.3	ref	
	Daily vaper	79.8	**4.81 (3.43–6.73)**	**< 0.001**	63.3	0.87 (0.64–1.18)	0.38	39.7	**4.52 (3.23–6.34)**	**<0.001**	51.3	**3.53 (2.58–4.82)**	**< 0.001**
	Non‐daily vaper	63.1	**2.79 (1.99–3.91)**	**< 0.001**	64.0	1.31 (0.94–1.84)	0.11	42.5	**5.07 (3.52–7.29)**	**<0.001**	36.0	**2.41 (1.69–3.44)**	**< 0.001**
	Trier	63.3	**2.44 (1.81–3.29)**	**< 0.001**	70.1	**1.44 (1.06–1.97)**	**0.021**	26.5	**2.51 (1.76–3.58)**	**<0.001**	34.4	**1.85 (1.34–2.55)**	**< 0.001**
	Ex‐vaper	56.3	**1.77 (1.29–2.41)**	**< 0.001**	65.8	1.20 (0.87–1.66)	0.26	25.0	**2.21 (1.52–3.22)**	**<0.001**	32.1	**1.75 (1.24–2.46)**	**0.001**
Income				**0.002**			**0.002**			0.55			**0.13**
	Low	54.3	ref		58.8	ref		22.3			32.3	ref	
	Moderate	59.1	1.19 (0.89–1.59)	0.25	63.4	1.25 (0.94–1.67)	0.13	27.0	1.22 (0.88–1.70)	0.23	31.3	0.97 (0.71–1.31)	0.83
	High	60.4	1.14 (0.86–1.52)	0.35	67.7	**1.61 (1.21–2.14)**	**0.001**	26.2	1.03 (0.75–1.43)	0.84	34.2	1.11 (0.83–1.49)	0.49
	Not disclosed	44.0	**0.57 (0.38–0.86)**	**0.008**	55.4	0.91 (0.61–1.36)	0.65	23.6	1.02 (0.64–1.62)	0.94	25.5	0.67 (0.43–1.05)	0.083
**Nicotine knowledge**													
Health risk from nicotine	Inaccurate response	54.8	ref		61.0	ref		24.9	ref		29.3	ref	
	Accurate (none or very small)	77.1	**1.82 (1.24–2.66)**	**0.002**	82.8	**2.29 (1.52–3.44)**	**< 0.001**	29.7	1.11 (0.77–1.59)	0.58	55.2	**2.15 (1.54–2.99)**	**< 0.001**
Causes most of the cancer	Inaccurate response (true)	46.2	ref		51.4	ref		25.5	ref		23.0	ref	
	Accurate response (false)	64.4	**1.79 (1.44–2.22)**	**< 0.001**	71.2	**2.05 (1.66–2.54)**	**< 0.001**	25.4	0.88 (0.69–1.12)	0.30	38.1	**1.68 (1.33–2.13)**	**< 0.001**

OR = odds ratio; CI = confidence interval; NRT = nicotine replacement therapy; EC = electronic cigarettes; *n* = 1720. (i) *R* ^2^ = 0.123 (Cox & Snell), 0.165 (Nagelkerke). Model χ^2^_(17)_ = 226.19, *P* < 0.001. (ii) *R* ^2^ = 0.074 (Cox & Snell), 0.102 (Nagelkerke). Model χ^2^_(17)_ = 132.90, *P* < 0.001. **(**iii) *R* ^2^ = 0.076 (Cox & Snell), 0.112 (Nagelkerke). Model χ^2^_(17)_ = 135.97, *P* < 0.001. (iv) *R* ^2^ = 0.098 (Cox & Snell), 0.138 (Nagelkerke). Model χ^2^_(17)_ = 178.30, *P* < 0.001. Bold type = *P <* 0.05; ref = reference category.

For the relative harm of NRT and tobacco cigarettes, 1.9% responded that NRT was more harmful than tobacco cigarettes, 15.2% responded equally harmful, 63.4% responded less harmful and 19.5% responded that they did not know. In the multivariable analysis, being aged 25–34 years was associated with lower odds, whereas trying vaping or having a high income were associated with greater odds, of rating NRT as less harmful than tobacco cigarettes versus all other outcomes (Table 3).

When asked about the relative addictiveness of EC compared to tobacco cigarettes, 4.7% responded that EC are more addictive, 49.0% responded equally addictive, 25.4% responded less addictive and 20.9% responded that they did not know. Being male, or having ever rather than never vaped, were associated with higher odds of responding that EC are less addictive than tobacco cigarettes versus all other outcomes in adjusted analyses (Table 3).

For the change in harm of stopping tobacco cigarettes and using EC instead, 32.2% responded that this would reduce health harms a great deal, 33.0% responded that this would reduce health harms a little, 13.0% responded this would not change harm at all, 2.8% responded this would increase health harms a little, 1.5% responded this would increase health harms a great deal and 17.5% did not know. In adjusted analyses, being aged 25–54 was associated with lower odds, and having ever vaped compared to never vaped was associated with higher odds, of responding that it would reduce health harms a great if a smoker stopped smoking cigarettes and started using EC instead (Table 3).

Accurate nicotine knowledge was associated with greater odds of responding that: EC are less harmful than tobacco cigarettes; NRT are less harmful than tobacco cigarettes; health harms would reduce a great deal if a smoker stopped smoking and used EC instead. Accurate nicotine knowledge was not significantly associated with the odds of responding that EC are less addictive than tobacco cigarettes (Table 3).

### Perceived main harms of EC

The top five concerns for participants around EC (at least one‐third of the sample) were: ‘not enough research has been done to understand all the possible harms’; ‘chemicals in the liquid might be harmful’; ‘not enough quality control or regulation, ‘you don't know exactly what you are getting’; ‘they may be addictive’; and ‘they may explode or catch fire’ (Table 4).

**Table 4:** Summary of ‘yes’ responses to questions around the perceived main harms of EC.

	% ‘Yes’ response to perceived concerns with EC (n)						
Perceived main harms of EC	Total % yes (n = 1720)	Daily vaper (n = 302)	Non‐daily vaper (n = 214)	Vaping trier (n = 294)	Ex‐vaper (n = 240)	Never vaped (n = 670)	χ^2^ (d.f.), P
There has not been enough research done to understand all the possible harms	48.3 (830)	46.0 (139)	37.9^b^ (81)	51.7 (152)	47.9 (115)	51.2 (343)	**χ** ^**2**^_**(4)**_ **= 13.61, *P* =** 0**.009**
The chemicals in the liquid might be harmful	41.6 (715)	33.4^b^ (101)	34.1^a^ (73)	49.3^a^ (145)	43.8 (105)	43.4 (291)	**χ** ^**2**^_**(4)**_ **= 21.81, *P* <** 0**.001**
There is not enough quality control or regulation—you don't know exactly what you are getting	37.8 (650)	24.5^b^ (74)	27.1^b^ (58)	48.0^b^ (141)	36.3 (87)	43.3^b^ (290)	**χ** ^**2**^_**(4)**_ **= 54.85, *P* <** 0**.001**
They may be addictive	33.3 (572)	32.1 (97)	29.4 (63)	32.3 (95)	37.5 (90)	33.9 (227)	χ^2^_(4)_ = 3.76, *P* = 0.44
They may explode or catch fire	36.9 (572)	24.8^b^ (75)	21.5^b^ (46)	41.5^b^ (122)	34.2 (82)	33.3^a^ (247)	**χ** ^**2**^_**(4)**_ **= 36.00, *P* <** 0**.001**
They reinforce the smoking habit	30.2 (520)	19.9^b^ (60)	22.9^a^ (49)	32.7 (96)	33.3 (80)	35.1^b^ (235)	**χ** ^**2**^_**(4)**_ **= 30.20, *P* <** 0**.001**
They may cause breathing/respiratory problems	23.4 (402)	19.2 (58)	18.2 (39)	28.2^a^ (83)	28.3 (68)	23.0 (154)	**χ** ^**2**^_**(4)**_ **= 13.32, *P* =** 0**.01**
The nicotine	22.9 (394)	24.2 (73)	22.0 (47)	22.8 (67)	24.6 (59)	22.1 (148)	χ^2^_(4)_ = 1.02, *P* = 0.91
They may cause cancer	14.5 (250)	10.9 (33)	10.8 (23)	14.3 (42)	14.6 (35)	17.5^a^ (117)	**χ** ^**2**^_**(4)**_ **= 10.27, *P* =** 0**.036**
They may cause heart disease	8.8 (152)	6.6 (20)	7.5 (16)	8.8 (26)	12.1 (29)	9.1 (61)	χ^2^_(4)_ = 5.53, *P* = 0.24
There are no harms	2.8 (49)^c^	–	–	–	–	–	**–**

Response options ‘yes’ or ‘no’. Bold type = *P* < 0.05.

a Indicates an adjusted standardized residual is greater than 2 and

b indicates an adjusted standardized residual greater than 3.

c This variable had a sample size below 50, so χ^2^ analyses were not conducted, as there was insufficient cell counts across the vaping categories. EC = electronic cigarettes; d.f. = degrees of freedom.

Perceived main harms differed significantly by vaping status for most items (Table 4). A lower percentage of non‐daily vapers endorsed that there had not been enough research to understand all possible harms. There was a lower percentage of daily and non‐daily vapers who endorsed that the chemicals in the liquid might be harmful; there is not enough quality control or regulation; EC may explode or catch fire; and EC may reinforce the smoking habit. A higher percentage of triers endorsed that the chemicals in the liquid might be harmful; not enough quality control or regulation; EC may explode or catch fire; and EC may cause breathing or respiratory problems. A higher percentage of never vapers endorsed that there is not enough quality control or regulation; EC may explode or catch fire; EC may reinforce the smoking habit; and EC may cause cancer. There was no significant difference based on vaping status to being concerned about nicotine, that EC may be addictive or that they may cause heart disease.

## Discussion

### Main findings

The first aim of this study was to investigate nicotine knowledge and perceptions of harm of EC and NRT relative to smoking, addictiveness of EC relative to smoking and change in harm to user if smoking was replaced with EC. Relative harm misperceptions of EC, NRT and tobacco cigarettes were common, with only approximately half believing accurately that EC were less harmful and just fewer than two‐thirds believing accurately that NRT was less harmful than tobacco smoking. Approximately a quarter perceived EC as less addictive than cigarettes.

The second aim was to see the associations of these perceptions with respondent characteristics, including nicotine knowledge. Perceptions mainly differed by vaping status; those who had never vaped were more likely to have misperceptions about the relative harm of EC and NRT compared with tobacco cigarettes. There was also some variation based on smoking status, age, gender and income. Knowledge of nicotine was poor: just fewer than nine of 10 smokers and ex‐smokers misattributed a greater portion of the risk in smoking to nicotine, and nearly four of 10 wrongly believed that nicotine is what causes cancer from smoking. Both inaccuracies were more common in those who had never vaped and those over 65 years. Inaccurate nicotine knowledge was associated with greater misperceptions of the relative harm of EC, NRT and tobacco cigarettes; however, inaccurate nicotine knowledge was not associated with perceiving EC as less addictive relative to tobacco cigarettes.

The third aim of the study was to explore main concerns around EC. The main perceived concerns around EC were that more research, regulation and quality control were needed on EC and concerns around chemicals in the liquid. These concerns were more likely among those who had never used EC, or only tried them, as opposed to daily and non‐daily vapers.

### What the results add to the literature

The results provide greater detail on perceptions of relative harm and the correlates of harm perception among a general population sample of UK smokers and ex‐smokers. Given that vaping status was associated with harm perceptions, it is possible that those who do not vape may not be using EC due to concerns about potential harms. This is in line with previous findings that fewer concerns about EC harms predicted EC use 9, and also supports previous research which found that users of nicotine products such as EC and NRT are more likely to correctly perceive relative harmfulness 7, 9. Alongside earlier data from the same longitudinal survey 9, which showed a decline in the proportion perceiving EC to be less harmful than cigarettes between 2012 and 2014, the present findings suggest that this trend of increased misperception has continued. It is unclear whether these misperceptions have been impacted by the revised EU TPD. This paper provides new information about NRT perceptions which, despite being licensed medications and being on the market for decades, are still perceived by more than a third of smokers and ex‐smokers as not less harmful than cigarettes.

Findings on inaccurate nicotine knowledge were in line with previous research, such as Siahpush *et al*. 8, who reported inaccurate understanding around links between nicotine and cancer. The results suggest that nicotine misperceptions may be related to misperceptions concerning the relative harmfulness of EC, NRT and smoking. Correcting misperceptions concerning nicotine may therefore help with understanding about the relative risks of different nicotine delivery products. Addictiveness perceptions of EC versus smoking were not, however, related to inaccurate knowledge. Perceptions have not always been found to be associated with usage 10, as there may be a complex interplay at work between accurate knowledge, harm perceptions and usage. Further research should examine in detail beliefs concerning nicotine and their role in harm perceptions.

### Limitations

This study recruited individuals who smoke currently, or used to smoke; it does not include the perspective of the small group of those who vape but have never smoked 4, 5. In addition, participants in this study may differ from the general population of smokers, as those who self‐select into research may have different views to those who do not choose to participate in research. Also, the relative harm perception items do not differentiate between harm to the smoker/ex‐smoker and others around the individual. Future research should consider the perspective of only‐vapers and differentiate harm perceptions to smokers, ex‐smokers and never‐smokers in future research. In particular, this sample had more older participants and fewer younger ones, so it would be useful to conduct further research looking at the harm perceptions of younger smokers and vapers. In addition, due to attrition, the sample may not be representative; however, as it was a large sample, there was still a good spread across gender, income, smoking and vaping status. Despite adjustment for key variables, a potential for unmeasured confounding remains, e.g. one variable which may reasonably be expected to be associated with harm perception is experience of smoking‐related illness by the respondent themselves or those close to them.

### Implications

It would be useful to explore why these misperceptions exist. This may be influenced by conflicting media reports on the topic and prominent reporting of malfunctioning devices, while fewer headlines are made by the aproximately 1500 people who die from smoking‐related illness every week in England alone 4. It might also be that the increasing pressure to regulate e‐cigarettes may be contributing to the perception that ‘they are more harmful’.

Furthermore, while many participants had inaccurate perceptions, a substantial number of participants replied they did not know, highlighting the need for more education and awareness. Misperceptions of the relative harms of EC and NRT compared with smoking tobacco cigarettes need to be targeted in public awareness campaigns and policy. In particular, public awareness campaigns should differentiate the role of smoke constituents other than nicotine in causing the main health harms (cancer, heart attacks, stroke) from the role of nicotine in continuing the addiction yet not having the same main health harms. Future research should also explore change mechanisms in harm perceptions to understand how to reverse the shifts in harm perceptions, and move towards more accurate understanding in the community. Accurate understanding is likely to assist smokers in making informed decisions around smoking, EC use and NRT in the aim to reduce health harms related to smoking tobacco cigarettes.

## Conclusion

The relative harms of EC and NRT compared to smoking tobacco cigarettes and the role of nicotine in the main health harms of smoking were overestimated by large proportions of smokers and ex‐smokers. These misperceptions have increased over time, and those who have never vaped are more likely to have misperceptions about relative harmfulness. Inaccurate nicotine knowledge was associated with increased relative harm misperceptions of EC and NRT compared to tobacco cigarettes.

## Declaration of interests

None.

## Supporting information


**Table S1** Demographic information and recoding of variables (wave 5).
