# Supplementary material for: Harm perceptions of e‐cigarettes and other nicotine products in a UK sample
Source: Addiction. 2019 Jan 3;114(5):879–88. doi: 10.1111/add.14502 (PMC6491935; doi:10.1111/add.14502)
Supplement: Supplementary file 1 — Table S1 Demographic information and recoding of variables (wave 5). [file ADD-114-879-s001.docx]

**Wilson S, Partos T, McNeill A, Brose LS. Harm perceptions of e-cigarettes and other nicotine products in a UK sample. Addiction, doi:10.1111/add.14502**

**Supplementary table**

Table S1 *Demographic information and recoding of variables (wave 5)*

| Variable | Question | Response options | Recoding |
| --- | --- | --- | --- |
| Gender | What is your gender? | Male; female | - |
| Age | What is your date of birth (year and month)? | Year and month used to create continuous variable | In years: 18-24; 25-34; 35-44; 45-54; 55-64; 65+. |
| Smoking status | Could you please tell us which of the following best applies to you now? | 1) I smoke cigarettes (including hand-rolled) every day; 2) I smoke cigarettes (including hand-rolled), but not every day; 3) I do not smoke cigarettes at all, but I do smoke tobacco of some kind (e.g. pipe or cigar); 4) I have stopped smoking completely since May-June 2016; 5) I stopped smoking completely before the last survey in May/June 2016 | daily smoker (1); non-daily smoker (2 and 3); ex-smoker (4 and 5) |
| Vaping status | Could you please tell us which of the following best applies to you now? | 1) I currently vape/use e-cigarettes daily; 2) I currently vape/use e-cigarettes but not every day 3) I have tried vaping/an e-cigarettes once or a few times; 4) I stopped vaping/using e-cigarettes since the previous survey in May/June 2016; 5) I have stopped vaping/using e-cigarettes since before the previous survey in May/June 2016; 6) I have never vaped/used e-cigarettes | daily vaper (1); non-daily vaper (2) trier (3); ex-vaper (4 and 5); and never vaped (6) |
| Income | Which of the following categories best describes your annual household income, that is the total income before taxes, or gross income, of all persons in your household combined, for one year? | under £6,500; £6,501-£15,000; £15, 001-£30,000; £30,001-£40,000; £40,001-£50,000; £50,001-£65,001; £65,001-95,001; £95,001 and over; prefer not to say; don’t know | low (under £15,000); moderate (£15,001 - £30,000); high (over £30,000); and not disclosed |
